# Distinct p21 requirements for regulating normal and self-reactive T cells through IFN-γ production

**DOI:** 10.1038/srep07691

**Published:** 2015-01-09

**Authors:** Lidia Daszkiewicz, Cristina Vázquez-Mateo, Gorjana Rackov, André Ballesteros-Tato, Kathrin Weber, Adrián Madrigal-Avilés, Mauro Di Pilato, Arun Fotedar, Rati Fotedar, Juana M. Flores, Mariano Esteban, Carlos Martínez-A, Dimitrios Balomenos

**Affiliations:** 1 Department of Immunology and Oncology, Centro Nacional de Biotecnología/CSIC, UAM Campus de Cantoblanco, E-28049 Madrid, Spain; 2 Department of Cellular and Molecular Biology, Centro Nacional de Biotecnología/CSIC, UAM Campus de Cantoblanco, E-28049 Madrid, Spain; 3 Cancer Cell Biology Program, Sidney Kimmel Cancer Center, San Diego, CA, USA; 4 Sanford-Burnham Medical Research Institute, San Diego, CA, USA; 5 Animal Biology Department, School of Veterinary Medicine, Universidad Complutense, Madrid, Spain

## Abstract

Self/non-self discrimination characterizes immunity and allows responses against pathogens but not self-antigens. Understanding the principles that govern this process is essential for designing autoimmunity treatments. p21 is thought to attenuate autoreactivity by limiting T cell expansion. Here, we provide direct evidence for a p21 role in controlling autoimmune T cell autoreactivity without affecting normal T cell responses. We studied C57BL/6, C57BL/6/*lpr* and MRL/*lpr* mice overexpressing p21 in T cells, and showed reduced autoreactivity and lymphadenopathy in C57BL/6/*lpr*, and reduced mortality in MRL/*lpr* mice. p21 inhibited effector/memory CD4^+^ CD8^+^ and CD4^−^CD8^−^ *lpr* T cell accumulation without altering defective *lpr* apoptosis. This was mediated by a previously non-described p21 function in limiting T cell overactivation and overproduction of IFN-γ, a key lupus cytokine. p21 did not affect normal T cell responses, revealing differential p21 requirements for autoreactive and normal T cell activity regulation. The underlying concept of these findings suggests potential treatments for lupus and autoimmune lymphoproliferative syndrome, without compromising normal immunity.

p21 (WAF1) is known mainly for its cell cycle inhibitor properties; it regulates early G1-S transition by inhibiting cyclin-dependent kinases in complex with cyclins A and E or D^1^. It was initially assumed that p21 deletion would lead to extensive tumor development but p21-deficient mice are essentially cancer-free^2^,^3^. Deficiency in p21 combined with mild autoreactive backgrounds such as 129/Sv × C57BL/6^4^ or the Gadd45a-deficient mice show severe lupus-like autoimmunity glomerulonephritis, which leads to death^5^,^6^. p21^−/−^ mice on the autoimmunity-resistant C57BL/6 (B6) background exhibited mild autoimmune manifestations^7^ and it was suggested that p21 acts as a suppressor of autoimmunity. In one report, lack of p21 appeared to reduce disease in autoimmune BXSB male background^8^, and it was considered that this controversy was probably due to the atypical BXSB background^7^,^9^. The p21 autoimmunity-suppressing activity was reinforced by analysis of Egr-2 deficient autoreactivity-developing mice, which downmodulate p21 expression in T cells^9^.

Data from p21−/− mice suggested a possible role for p21 in the expansion of activated but not of naïve T cells^7^. In a different system, increased p21 expression by CD4^+^ T cells from elite (infection-free) HIV-exposed individuals, appeared critical for evasion of HIV infection^10^. In addition to regulating adaptive immune responses, p21 controls innate immunity, modulating macrophage activation through the NF-κB activation pathway^11^ and inflammatory cytokine production^11^,^12^,^13^. p21 thus emerges as an important regulator of immunity that controls innate and adaptive responses, and maintains autoimmunity development at bay^14^,^15^,^16^.

*lpr* (lymphoproliferation spontaneous mutation) mice deficient in Fas (CD95), show defective activation-induced cell death (AICD) of *in vitro* restimulated T cells^17^. *lpr* mice develop lymphadenopathy due to accumulation of double negative T cells (DN; TCRαβ^+^CD4^−^CD8^−^B220^+^), and lupus-like autoimmune disease, probably due to CD4^+^ T cell hyperactivation^18^. One of the unexplained symptoms caused by Fas deficiency is massive hyperproliferation of DN T cells, CD4^+^ effector (CD44^hi^/CD62L^hi^), memory (CD44^hi^/CD62L^lo^), and CD8^+^ effector/memory T cells in lymphoid organs. Accumulation of effector/memory T cells is critical for development of autoimmunity, as they secrete large amounts of IFN-γ, a cytokine necessary for lupus development in *lpr* and other spontaneous or induced murine lupus models^19^,^20^,^21^,^22^. C57BL/6/*lpr* (B6/*lpr*) mice develop anti-DNA antibodies and mild, non-lethal glomerulonephritis, whereas *lpr* mice on the autoimmune-prone MRL background (MRL/*lpr*) show severe autoimmune manifestations that lead to kidney failure and death^18^. Humans with defective Fas/Fas ligand (FasL) signaling develop autoimmune lymphoproliferative syndrome (ALPS)^23^,^24^,^25^, whose main characteristics are DN T cell accumulation and lymphadenopathy development, propensity to lupus autoimmunity, and increased risk for lymphoma.

To determine a possible therapeutic role for p21 in lupus autoimmunity and to provide direct evidence for an effect of p21 on autoreactive T cells, we analyzed the impact of T cell-directed overexpression of p21 in B6, B6/*lpr* and MRL/*lpr* mice. We found that p21 overexpression inhibited B6/*lpr* DN T cell lymphadenopathy and decreased effector/memory T cell expansion and autoimmune symptoms. Further analysis revealed an unanticipated p21 capacity to decrease the activation of effector/memory B6/*lpr* T cells and their IFN-γ production. p21 is a potent autoimmunity suppressor, since when overexpressed in MRL/*lpr* mice, efficiently reduced death rates. Exogenous p21 effects were evident in *lpr* but not in control B6 mice, indicating that autoimmune but not normal T cells require p21 to control activation and IFN-γ production. Therefore, therapeutic approaches that target autoimmunity but not normal responses are feasible.

## Results

### T cell-directed p21 expression inhibits effector/memory T cell accumulation in B6/*lpr* but not in B6 mice

By two months of age, B6/*lpr* mice show a predisposition to autoimmunity and begin to accumulate memory and DN T cells in lymphoid organs, with development of autoimmune characteristics and lymphadenopathy^17^. As lack of p21 leads to increased expansion of repeatedly stimulated T cells *in vitro* without affecting primary T cell responses^7^, we hypothesized that directed transgenic p21 expression in B6/*lpr* mouse T cells would reduce spontaneous accumulation of effector/memory T cells and ameliorate lupus characteristics in these mice. We generated B6 and B6/*lpr* mice that specifically express a human p21 transgene in T cells under the proximal Lck promoter (B6-p21tg and B6/*lpr*-p21tg), and studied the *in vivo* characteristics of peripheral T cells. T cell development and differentiation in B6-p21tg mice is normal^26^, as confirmed by similarity of thymic populations in B6 and B6-p21tg and in B6/*lpr* and B6/*lpr*-p21tg mice (Fig. S1).

**Figure 1:**
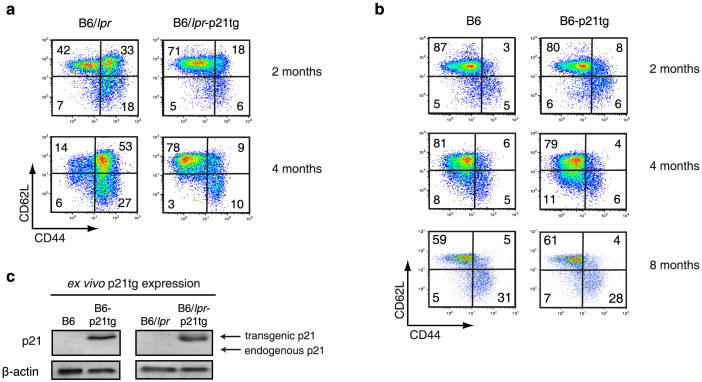
p21 overexpression inhibits age-dependent accumulation of B6/*lpr* effector and memory T cells. (a) Flow cytometry of lymph node CD4^+^ T cells at 2 and 4 months of age showed reduced accumulation of B6/*lpr*-p21tg CD44^hi^CD62L^hi^ and CD44^hi^CD62L^lo^ subsets compared to B6/*lpr* T cells. Values show mean from one representative experiment (*n* = 4 mice; p < 10^−5^ for both subsets) of 3 performed. (b) Similar proportions of CD44^hi^CD62L^hi^ and CD44^hi^CD62L^lo^ CD4^+^ T cells in B6 and B6-p21tg mouse lymph nodes at 2, 4 and 8 months of age. Values show mean from one representative experiment (n = 4 mice) of 3 performed. (c) Western blot showed high p21 transgene expression in sorted naïve CD4^+^ cells from B6-p21tg and B6/*lpr*-p21tg mice. β-actin was used as a loading control. The samples were derived from the same experiment and the two gels were run and processed simultaneously under the same experimental conditions.

Definition of effector and memory T cells is complex, and several subgroups of these cell populations have been described^27^; here we use the basic definition of effector (CD44^hi^/CD62L^hi^) and memory (CD44^hi^/CD62L^lo^) CD4^+^ T cells. These two cell groups expand *in vivo* and accumulate with age in B6 mice, possibly due to exposure to endogenous and environmental antigens. In B6/*lpr* mice, however, effector/memory CD4^+^ T cells accumulate to a much greater extent, as confirmed at two and four months of age (Fig. 1A, B, left). Enhanced p21 expression in T cells greatly reduced effector and memory CD4^+^ T cell accumulation in B6/*lpr*-p21tg mice at two months, and more so at four months of age as compared to B6/*lpr* mice (Fig. 1A). The transgene nonetheless did not affect the percentage of effector and memory T cells in B6-p21tg mice (Fig. 1B). Even at eight months of age, when effector and memory T cells accumulate in larger proportions, we found no difference between B6 and B6-p21tg mice (Fig. 1B). CD4^+^ T cells from two-month-old B6-p21tg and B6/*lpr*-p21tg mice expressed high p21 transgene levels, while endogenous p21 was not detected in these or the equivalent B6 and B6/*lpr* cells (Fig. 1C). The human p21 transgene appears as a higher molecular weight band as compared to that of the endogenous p21. The expression of the p21 transgene justified its effect in lowering effector and memory B6/*lpr*-p21tg T cells. The results indicate that *lpr* T cells require enhanced p21 expression to control T cell activation and memory levels effectively, whereas exogenous p21 is not needed to regulate B6 T cell activation.

### p21 overexpression reduces B6/*lpr* CD4^+^ T cell activation and IFN-γ production

Enhanced p21 expression severely reduced the *lpr* effector/memory CD4^+^ T cell population, which is large in these mice. We tested whether p21 overexpression affects activation of effector/memory T cells in B6/*lpr*-p21tg mice, and whether production of inflammatory cytokines such as IFN-γ was compromised in this reduced cell population.

**Figure 2:**
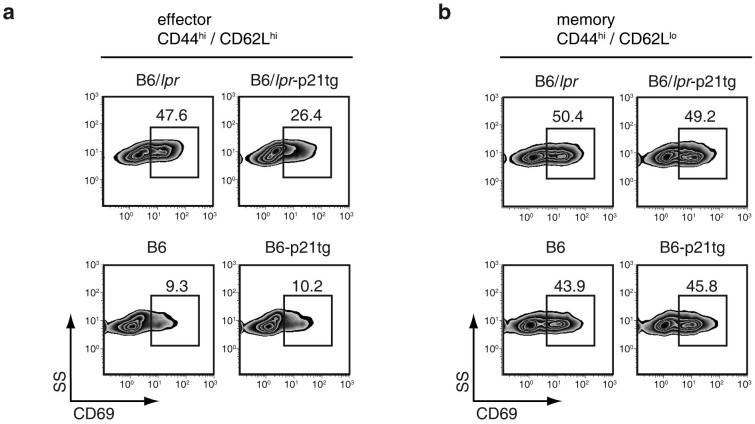
p21 overexpression reduces activation of B6/*lpr*-p21tg CD4^+^ T cells. Lymph node CD44^hi^CD62L^hi^ (effector) and CD44^hi^CD62L^lo^ (memory) CD4^+^ T cells from 4-month-old B6/*lpr*, B6/*lpr*-p21tg, B6 and B6-p21tg mice were analyzed by flow cytometry. (a) Reduced CD69 expression in B6/*lpr*-p21tg CD44^hi^CD62L^hi^ CD4^+^ T cells compared with those from B6/*lpr* mice. Data are from one representative experiment (*n* = 4 mice; p < 10^−4^) of 2 performed. CD69 levels were similar on B6-p21tg and B6 CD44^hi^CD62L^hi^ CD4^+^ T cells. (b) Similar CD69 expression for B6/*lpr*-p21tg and B6/*lpr* CD44^hi^CD62L^lo^ CD4^+^ T cells and the equivalent B6-p21tg and B6 cell populations. Data from one representative experiment of two performed (*n* = 4 mice each).

CD69 is a major T cell activation marker and is associated with memory T cells^28^. Effector *lpr* T cells in lymph node showed greatly increased CD69 levels (>4-fold) compared to the equivalent B6 population (Fig. 2A, left). This suggested increased cell activation and corroborated the greatly augmented effector T cell population in B6/*lpr* mice. In B6/*lpr*-p21tg mice, overexpressed p21 reduced CD69 expression by ~50% in the effector T cell population (Fig. 2A, top), whereas exogenous p21 had no effect on the high CD69 levels by memory B6/*lpr* T cells (Fig. 2B, top). Enhanced p21 expression thus efficiently reduced not only the elevated percentage of B6/*lpr*-p21tg effector CD4^+^ T cells, but also their activation, as implied by sharply reduced CD69 expression. Overexpressed p21 had no effect on CD69 expression by B6 lymph node effector and memory T cells (Fig. 2A, B, bottom panels). The data show that exogenous p21 affected mainly CD69-overexpressing effector B6/*lpr* T cells, indicating that p21 targets the hyperactivated T cell population.

**Figure 3:**
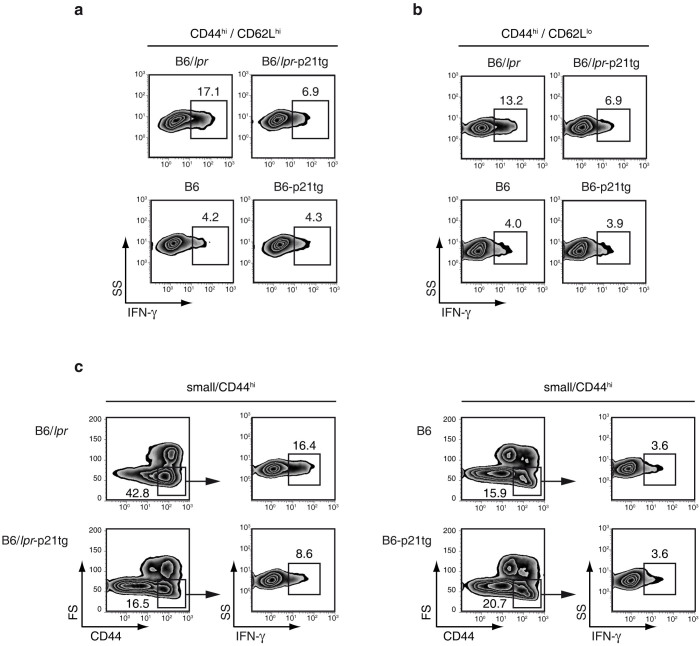
p21 overexpression reduces IFN-γ production in B6/*lpr*-p21tg effector and memory CD4^+^ T cells. Lymph node CD4^+^ T cells from 4-month-old B6/*lpr*, B6/*lpr*-p21tg, B6 and B6-p21tg mice were analyzed by flow cytometry. (a) Intracellular cytokine analysis showed reduced frequency of IFN-γ-producing cells in CD44^hi^CD62L^hi^ CD4^+^ T cell populations from B6/*lpr*-p21tg compared with B6/*lpr* mice. Data are from one representative experiment (*n* = 4 mice; p < 10^−2^) of 2 performed. Percentages of IFN-γ-producing cells were similar for B6-p21tg and B6 CD44^hi^CD62L^hi^ CD4^+^ T cells. (b) The frequency of IFN-γ-producing CD44^hi^CD62L^lo^ CD4^+^ T cells was lower in B6/*lpr*-p21tg compared with B6/*lpr* mice. Data are from one representative experiment (*n* = 4 mice; p < 10^−2^) of 2 performed. Percentages of IFN-γ-producing CD44^hi^CD62L^lo^ CD4^+^ T cells were similar for B6-p21tg and B6 mice. (c) Small CD44^hi^ cells showed reduced frequency of IFN-γ-producing cells in B6/*lpr*-p21tg compared with B6/*lpr* mice. Percentages of IFN-γ-producing small CD44^hi^ CD4^+^ T cells were similar for B6-p21tg and B6 mice.

To determine whether overexpressed p21 interfered with B6/*lpr* T cell function, we measured inflammatory cytokine production in B6/*lpr* and B6/*lpr*-p21tg effector and memory T cells. We analyzed expression of IFN-γ, IL-17 and IL-2, which are associated with the *lpr* autoimmune phenotype^29^,^30^,^31^,^32^, and the degree of IL-4 secretion as a control. Expression of IFN-γ, which modulates anti-DNA antibody production and disease development in several lupus models^33^, was markedly increased by effector and memory CD4^+^ T cells in B6/*lpr* mice compared to B6 controls (Fig. 3A, B, left panels). Overexpressed p21 led to a sharp decrease in the fraction of IFN-γ-producing B6/*lpr*-p21tg effector and memory CD4^+^ T cells (Fig. 3A, B, top), but had no effect on the equivalent B6-p21tg T cells (Fig. 3A, B, bottom). Definition of memory T cells by the expression of CD44 and CD62L might be inexact, and characterization of memory T cells is complex. As *bona fide* memory T cells are defined as small CD44^hi^ cells (selected by forward/side scatter)^34^, we used this definition of memory T cells, and again found that p21 overexpression greatly reduced the percentage of IFN-γ-producing small B6/*lpr*-p21tg memory T cells (Fig. 3C, left), but had no effect on the equivalent B6-p21tg T cell population (Fig. 3C, right).

IL-17, another lupus-associated cytokine expressed by B6/*lpr* T cells^30^,^31^, was produced by a small number of CD4^+^ T cells (<3%) and was unaffected by p21 overexpression (not shown). IL-2 levels were substantially lower in B6/*lpr* memory T cells than in B6 controls (3.3% vs. 10.4%; Fig. S2), in accordance with previous reports^29^; the p21 transgene did not affect the difference in IL-2 expression (Fig. S2). IL-4 expression was minimal (<1%) in T cells from all mice tested (not shown).

The data indicate that the p21 transgene controls not only effector/memory T cell accumulation in B6/*lpr*-p21tg mice, but also regulates their intrinsic activation and IFN-γ production potential.

### The p21 transgene negatively regulates T cell hyperproliferation in B6/*lpr*-p21tg mice

**Figure 4:**
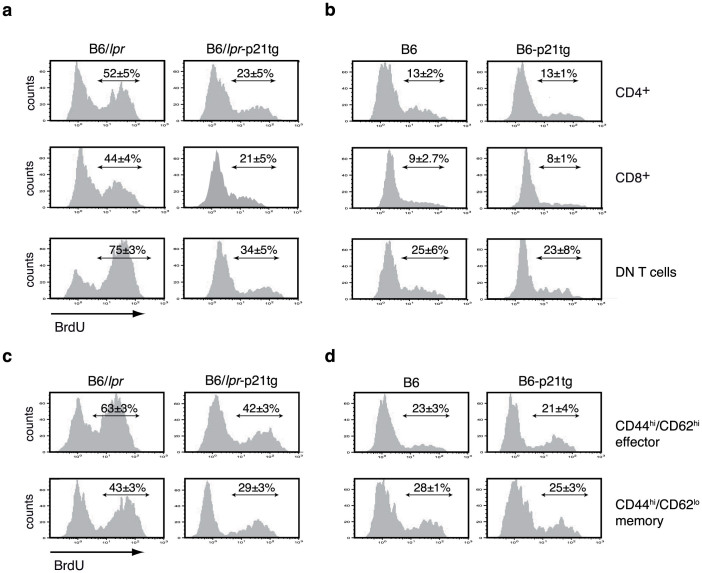
p21 overexpression reduces *in vivo* T cell hyperproliferation. (a) Transgenic p21 expression reduced *in vivo* BrdU incorporation by B6/*lpr* CD4^+^, CD8^+^, and DN T cells. Data show mean ± SD (n = 4 mice; p < 10^−5^ for the three T cell types). (b) Similar BrdU incorporation for control B6 and B6-p21tg CD4^+^, CD8^+^, and DN T cells. (c) p21 transgenic expression reduced *in vivo* BrdU incorporation by effector (CD44^hi^CD62L^hi^) and memory (CD44^hi^CD62L^lo^) B6/*lpr* T cells. (d) B6 and B6-p21tg effector and memory T cells showed similar *in vivo* BrdU incorporation. T cells were obtained from 2-month-old mice given BrdU in drinking water for 9 days. For (c) and (d) representative histograms are shown; mean ± SD (*n* = 4 mice).

An unexplained characteristic of the *lpr* mouse phenotype is the *in vivo* hyperproliferation of lymph node CD4^+^, CD8^+^ and DN T cells^19^,^20^, which is not directly clarified by the apoptosis defect observed *in vitro*. Here we used *in vivo* BrdU incorporation to confirm these results, showing hyperproliferation of B6/*lpr* CD4^+^, CD8^+^ and DN T cells compared to B6 controls (Fig. 4A, B, left panels). p21 overexpression greatly inhibited lymph node CD4^+^, CD8^+^ and DN T cell hyperproliferation in B6/*lpr*-p21tg mice (>2-fold, Fig. 4A), but had no effect on the *in vivo* proliferative characteristics of B6-p21tg T cells (Fig. 4B).

Analysis of BrdU incorporation showed much greater proliferation of effector and memory B6/*lpr* mouse T cells compared to B6 controls (63% vs. 23%, and 43% vs. 28%, respectively; Fig. 4C, D, left). Enhanced p21 expression had a major anti-proliferative effect on B6/*lpr*-p21tg CD4^+^ T cells (>68%) compared to B6/*lpr* effectors (Fig. 4C, top), with less extensive but significantly reduced proliferation in the equivalent memory T cell populations (28%) (Fig. 4C, bottom). Enhanced p21 expression had no influence on effector or memory T cell subsets in B6-p21tg mice (Fig. 4D).

Our results indicated that overexpressed p21 regulates hyperactivation, IFN-γ production and hyperproliferation of B6/*lpr*-p21tg effector/memory T cells, with no effect on the B6-p21tg T cells. These data suggest that p21 exerts its autoimmunity-suppressive activity by controlling the increased activity and proliferation of effector T cells.

### Elevated p21 expression does not modify the apoptotic defect of B6/*lpr* T cells

**Figure 5:**
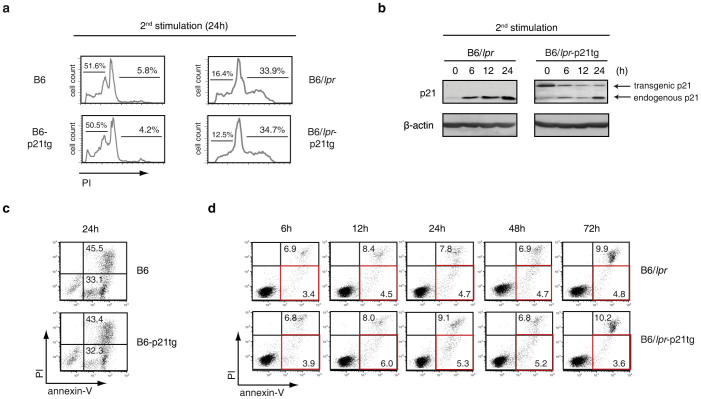
Apoptosis defect in B6/*lpr* T cells is unaffected by p21 overexpression. (a) Purified CD4^+^ T cells from B6, B6-p21tg, B6/*lpr* and B6/*lpr*-p21tg mouse spleens were ConA-stimulated and -restimulated after the IL-2 expansion phase. Cell cycle analysis at 24 h after restimulation showed similar levels of cell death (presence of an apoptotic hypodiploid peak in cell cycle profiles) in B6 and B6-p21tg T cells. Lack of the apoptotic peak was similar for B6/*lpr* and B6/*lpr*-p21tg T cells. Representative histograms are from three independent experiments. (b) Western blot analysis showing endogenous and transgenic p21 protein levels at the end of the IL-2 expansion period (0 h) and at several points after ConA restimulation of B6/*lpr* and B6/*lpr*-p21tg T cells. β-actin was used as a loading control. The samples were derived from the same experiment and the two gels were run and processed simultaneously under the same experimental conditions. (c) Annexin-V/propidium iodide (PI) double staining at 24 h after ConA restimulation showed similar levels of apoptotic (annexin-V^+^/PI^−^) and post-apoptotic (annexin-V^+^/PI^+^) B6 and B6/*lpr*-p21tg T cells. (d) Similar cell death defects in B6/*lpr* and B6/*lpr*-p21tg T cells at various times after ConA restimulation, as shown by percentages of apoptotic and post-apoptotic events. Apoptotic T cells are shown in red squares. (c, d) Data are representative of two independent experiments.

To determine whether the reduced hyperactivation and IFN-γ production as well as the expansion of B6/*lpr*-p21 effector/memory T cells were due to attenuation of the B6/*lpr* T cell apoptosis defect by the p21 transgene, we used the classical AICD protocol to compare B6/*lpr* and B6/*lpr*-p21 T cell death. After primary TCR stimulation with concanavalin A (ConA) and IL-2-dependent culture, B6, B6-p21tg, B6/*lpr* and B6/*lpr*-p21tg CD4^+^ T cells received a second stimulation, leading to activation of the Fas/FasL system and apoptosis. AICD was similar in B6 and B6-p21tg T cells after restimulation, as shown by a comparable apoptotic hypodiploid peak and proportion of death in cell cycle profiles (Fig. 5A, left). In these conditions, we found a similar defect in the B6/*lpr* and B6/*lpr*-p21tg T cells, with absence of the apoptotic hypodiploid peak due to the *lpr* AICD defect (Fig. 5A, right).

The results suggested that the p21 transgene had no effect on normal or defective AICD. This was confirmed by the observation that p21 transgene expression was high in B6-p21tg and B6/*lpr*-p21tg T cells during AICD induction. In B6/*lpr*-p21 T cells, the p21tg was highly expressed at the end of IL-2 treatment (Fig. 5B, 0 h). After secondary stimulation, expression was maintained at 6, 12 and 24 h, albeit at lower levels. Endogenous p21 expression was minimal at the end of IL-2 culture: it increased in B6/*lpr* T cells after secondary stimulation, and more moderately in B6/*lpr*-p21 T cells, probably due to the p21 transgene overexpression, resulting in lower endogenous p21 levels in B6/*lpr*-p21 T cells (Fig. 5B). Expression of transgenic and endogenous p21 was similar in B6-p21tg and B6 T cell cultures (Fig. S3).

We tested apoptosis induction, using annexin-V/propidium iodide (PI) staining to confirm the cell cycle analysis results. In B6 and B6-p21tg cultures, we observed similar frequencies of apoptotic (annexin-V^+^/PI^−^; 33.1% and 32.3%, respectively) and post-apoptotic dead T cells (annexin-V^+^/PI^+^; 45.5% and 43.4% respectively) (Fig. 5C). These data indicated that p21 overexpression did not influence normal Fas/FasL-dependent AICD. In similar experiments, we carried out annexin-V/PI staining of B6/*lpr* and B6/*lpr*-p21tg T cells; apoptosis was similarly defective for both cell types, as indicated by the small percentage of apopotic T cells (<6%) in a time course study (6 to 72 h). Percentages of necrotic cells were also low (<10%) and were similar in B6/*lpr* and B6/*lpr*-p21tg T cell cultures (Fig. 5D).

These results indicated that the p21 transgene is expressed at different times during the AICD process, and exclude the possibility that it acts as an apoptosis-promoting factor in B6 or in apoptosis-deficient B6/*lpr* T cells. Our findings suggest that the p21 transgene has an apoptosis-independent effect on B6/*lpr*-p21tg mouse effector/memory T cells that reduces their excessive accumulation, activation and IFN-γ production.

### p21 overexpression reduces B6/*lpr* T cell activation and proliferation after repeated *in vitro* stimulation

**Figure 6:**
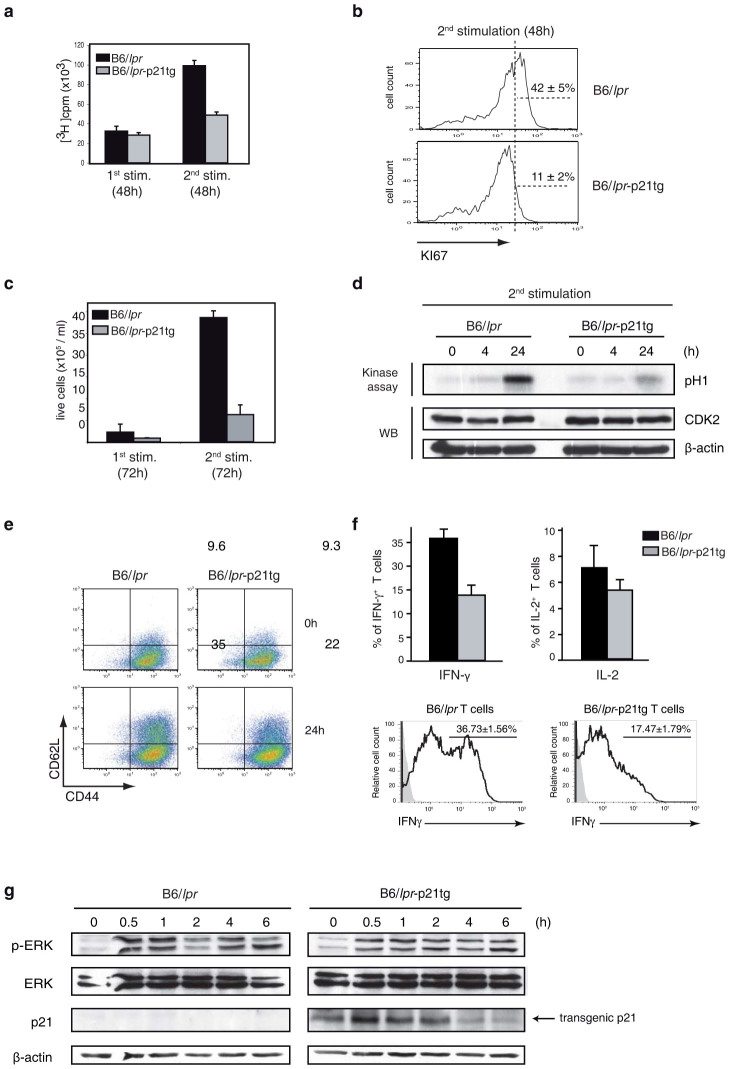
p21 overexpression decreases *in vitro* B6/*lpr* T cell hyperproliferation after secondary stimulation. CD4^+^ T cells from B6/*lpr* and B6/*lpr*-p21tg mouse spleens were ConA-stimulated (1^st^ stimulation) and -restimulated (2^nd^ stimulation) after the IL-2 expansion phase. (a) Transgenic p21 reduced T cell responses only after restimulation. Proliferation was measured by [^3^H]thymidine uptake 48 h after ConA stimulation or restimulation. Values show mean ± SD from one representative experiment (*n* = 4 T cell preparations from distinct mice; p < 4 × 10^−6^) of four performed. (b) Flow cytometry showed reduced expression of the Ki-67 proliferation marker in B6/*lpr*-p21tg compared to B6/*lpr* T cells after restimulation. Data from one representative experiment of three performed. (c) Absolute numbers of B6/*lpr*-p21tg T cells generated after restimulation were decreased compared to B6/*lpr* T cells. Live cells were determined by trypan blue exclusion. Values show mean ± SD from one representative experiment of three performed (*n* = 4 T cell preparations from distinct mice; p < 10^−7^). (d) CDK2 activity after ConA restimulation. Assay products were resolved in SDS-PAGE and phosphorylated histone (H1) was detected by autoradiography. Extracts were analyzed by Western blot for CDK2 levels as loading control. (e) Similar percentages of B6/*lpr* and B6/*lpr*-p21tg CD44^high^CD62L^high^ CD4^+^ T cells at the end of the IL-2 expansion (top). At 24 h after restimulation, the percentage of B6/*lpr*-p21tg CD44^high^CD62L^high^ cells was decreased compared to B6/*lpr* (bottom). (f) Flow cytometry of CD4^+^ T cells for intracellular cytokines. IFN-γ-producing B6/*lpr*-p21tg CD4^+^ T cells were reduced compared to B6/*lpr* 24 h after restimulation. Values show mean ± SD (n = 4 mice each; p < 10^−2^). Differences in IL-2 were not significant (top). After restimulation with IL-2, IL-12 and IL-18, IFN-γ-producing B6/*lpr*-p21tg were lower compared to B6/*lpr* T cells. Values show mean ± SD (n = 3 mice, p = 0.0013) (bottom). (g) Western blot showing ERK phosphorylation and transgenic p21 protein levels in B6/*lpr* and B6/*lpr*-p21tg T cells after restimulation. β-actin was used as a loading control. The samples were derived from the same experiment and the two gels were run and processed simultaneously under the same experimental conditions.

**Figure 7:**
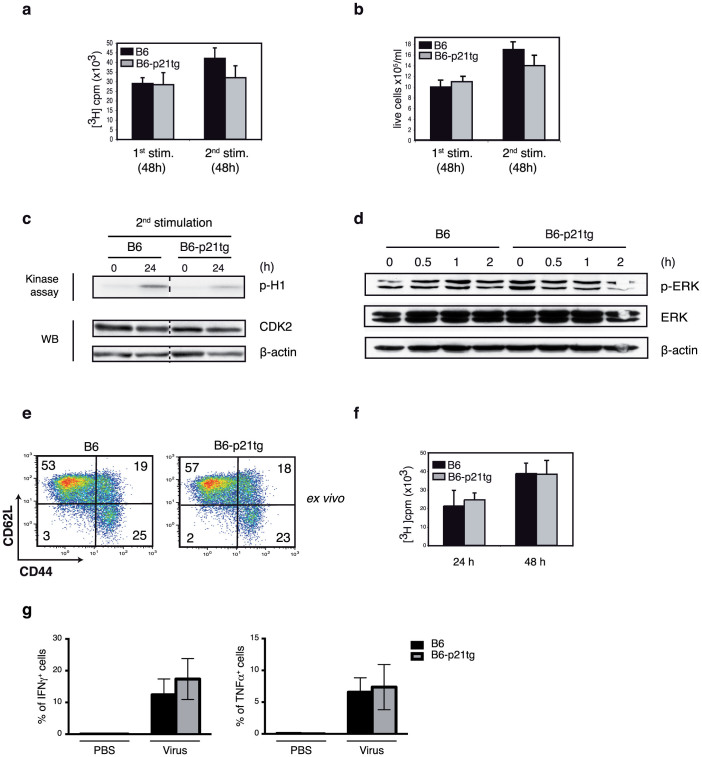
p21 overexpression does not affect B6 T cell proliferation or activation. CD4^+^ T cells from B6 and B6-p21tg mouse spleens were ConA-stimulated or -restimulated (in the presence of zVAD) after an IL-2 expansion phase. (a) Proliferation was measured by [^3^H]thymidine uptake at 48 h after first or second ConA challenge. Values show mean ± SD (*n* = 4 T cell preparations from distinct mice). (b) Generation of newly divided B6 and B6-p21tg T cells was similar after primary and secondary stimulation, as determined by trypan blue exclusion. Values show mean ± SD (*n* = 3 T cell preparations from distinct mice). (c) CDK2 activity after ConA restimulation. Assay products were resolved in SDS-PAGE, and phosphorylated histone (H1) was detected by autoradiography. Extracts were analyzed by Western blot for CDK2 as loading control. Dotted line represents cropping of single gels. Full-length gels are presented in Supplementary Figure 6. (d) ERK phosphorylation and transgenic p21 protein levels in B6 and B6-p21tg T cells at various times post-restimulation. β-actin was used as a loading control. (e) Lymph nodes from OVA-immunized and –boosted B6 and B6-p21tg mice were analyzed to quantify memory CD4^+^ T cells. (f) Purified CD4^+^ splenocytes were from OVA-immunized and -boosted B6 and B6-p21tg mice were cultured with OVA and irradiated splenocytes, and showed similar [^3^H]thymidine uptake at 24 and 48 h postactivation. Data are representative from two experiments (*n* = 4 mice each). (g) Splenocytes from vaccinia-injected B6 and B6-p21tg mice were cultured with vaccinia peptide after double *in vivo* immunization, and showed similar specific CD8 T-cell immune responses, as detected by the proportions of IFN-γ- and TNF-α-producing cells. The non-specific responses obtained in the absence of vaccinia peptide were substracted. Data show mean ± SD (*n* = 5 mice).

We performed *in vitro* stimulation studies to further examine the role of the p21 transgene as an attenuator of B6/*lpr*-p21tg T cell activation and proliferation. In a previous study of p21^−/−^ mice, we showed that p21 controls T cell expansion after secondary but not after primary stimulation^7^. Here we found reduced expansion and activation of B6/*lpr*-p21tg T cells after secondary stimulation, using a dual stimulation protocol with an intermediate IL-2 expansion step as described above for AICD induction. In these experiments, cultured B6/*lpr* and B6/*lpr*-p21tg T cells proliferated similarly after primary ConA stimulation, as measured by [^3^H]thymidine uptake (Fig. 6A). In contrast, we detected a sharp decrease (>50%) in [^3^H]thymidine uptake in B6/*lpr*-p21tg compared to B6/*lpr* T cells after secondary stimulation (Fig. 6A). Results were similar after anti-CD3 and -CD28 TCR stimulation of B6/*lpr* and B6/*lpr*-p21tg T cells (Fig. S4). Reduction of cell cycle progression due to p21 overexpression was further confirmed by >3-fold lower expression of the Ki-67 proliferation marker^35^ in B6/*lpr*-p21tg than in B6/*lpr* T cells after secondary stimulation (Fig. 6B). These findings were corroborated by a marked reduction (>5-fold) in the absolute number of T cells that divided in B6/*lpr*-p21tg *vs*. B6/*lpr* cultures at 72 h post-secondary activation, whereas there were no notable differences after primary stimulation (Fig. 6C). The p21 transgene did not significantly affect B6-p21tg T cell [^3^H]thymidine uptake or the number of divided T cells compared to B6 T cells after secondary or primary stimulation (Fig. 7A, B). To avoid Fas-dependent apoptosis of restimulated B6 and B6-p21tg T cells, we used the caspase inhibitor zVAD (benzyloxycarbonyl-Val-Ala-Asp-fluoromethylketone) in restimulation experiments. In B6/*lpr and* B6/*lpr*-p21tg secondary stimulation cultures, zVAD was used as a control to confirm that it did not influence the inhibitory effects of the p21 transgene.

We excluded the possibility that p21 overexpression in B6 T cells affected *in vivo* CD4^+^ T cell responses, since B6 and B6-p21tg mice immunized with OVA in CFA, followed 3 weeks later by OVA challenge in IFA, showed no differences in overall memory T cell generation (Fig. 7E). Purified CD4^+^ splenocytes from B6 and B6-p21tg mice obtained 7 days after a second OVA boost, cultured with OVA and irradiated splenocytes, showed a similar OVA response at 24 and 48 h postactivation (Fig. 7F). To further demonstrate that p21 does not affect normal immune T cell responses, we immunized B6 and B6-p21tg mice with vaccinia virus, an infection model that targets CD8^+^ T cells. We injected B6 and B6-p21tg mice with Modified Vaccinia Ankara (MVA) virus^36^ at day 0 and day 8 and immune responses were measured 10 days after the last dose. Splenocytes were stimulated with the MHC class I restricted B8R_20–27_ viral peptide^37^ and the response of CD8^+^ spleen T cells was measured by determining the proportion of IFN-γ- and TNF-α-expressing CD8^+^ T cells. We found similar responses for B6 and B6-p21tg CD8^+^ T cells after immunization and restimulation (Fig. 7G).

Our data show that, as in the *in vivo* studies, p21 limits expansion of B6/*lpr*-p21tg but not of B6-p21tg T cells in *in vitro* restimulation conditions. As p21 inhibits CDK2 and regulates proliferation, we examined CDK2 activity in B6/*lpr*-p21tg T cells after secondary stimulation; at 24 h post-stimulation, we found reduced CDK2 activity in B6/*lpr*-p21tg T cells (Fig. 6D), which could account for their decreased expansion. Nonetheless, CDK2 activity was reduced in B6-p21tg T cells compared to B6 counterparts (Fig. 7C), which showed similar proliferation capacity after restimulation (Fig. 7A, B). These results raise the possibility that reduction of CDK2 activity by p21 alone is insufficient to limit the increased responses of B6/*lpr* T cells.

In addition to its effect on *in vivo* hyperproliferation, the p21 transgene lowered T cell activation and IFN-γ production by B6/*lpr*-p21tg CD4^+^ T cells. In the dual stimulation protocol, IL-2-expanded B6/*lpr* and B6/*lpr*-p21tg CD4^+^ T cell populations had similar percentages (<10%) of effector cells with a CD44^hi^CD62L^hi^ phenotype, suggesting that the p21 transgene had no effect on the initial stimulation process (Fig. 6E, top). The remaining cells had a CD44^hi^CD62L^lo^ memory cell phenotype. After secondary stimulation of B6/*lpr* T cells (24 h), the proportion of effectors increased from 9.6% to 35%, suggesting that a fraction of cells reverted from memory to effector phenotype as a result of activation (Fig. 6E, left). In B6/*lpr*-p21tg cultures, conversion to effector T cells was clearly lower after restimulation (22%; Fig. 6E, right), reflecting a lower activation level. These data suggested that the p21 transgene negatively regulated B6/*lpr* T cell activation after secondary stimulation. In accordance with these results, a significantly smaller fraction of restimulated B6/*lpr*-p21tg T cells produced IFN-γ compared to B6/*lpr* T cells (Fig. 6F, top). The results coincided with our *in vivo* data showing a limited percentage of IFN-γ-producing T cells in B6/*lpr*-p21tg mice. We detected no significant differences for IL-2 production in equivalent *in vitro* experiments (Fig. 6F, top). In order to provide additional evidence for a role of p21 in reducing IFN-γ production, we employed a model were IFN-γ is directly induced. IL-12 directly drives IFN-γ production, which is enhanced by IL-18 in T cells during TCR activation^38^,^39^. In our case, T cells were initially stimulated with ConA, subsequently exposed to IL-2 and then triggered by IL-12, IL-18 and IL-2. Under these conditions, B6/*lpr* T cells showed a high proportion of IFN-γ positive cells (more than 36%), while p21 transgenic expression in B6/*lpr*-p21tg T cells strongly inhibited IFN-γ production (Fig. 6F, bottom). These data showed that enhanced p21 expression suppresses IL-12- and IL-18-dependent IFN-γ induction.

Activation anomalies corresponding to the amount of phosphorylated ERK (p-ERK) are thought to enhance autoreactivity and IFN-γ production^40^,^41^; we therefore examined the regulatory effect of the p21 transgene on ERK phosphorylation of B6/*lpr* and B6/*lpr*-p21tg T cells. Western blot analysis showed p-ERK variations in B6/*lpr*-p21tg compared to B6/*lpr* T cells, in a period up to 6 h after secondary stimulation. ERK phosphorylation was lower at 0.5 and 1 h post-stimulation, with higher p-ERK levels at 2 h post-activation in B6/*lpr*-p21tg cultures (Fig. 6G). An initial delay in ERK phosporylation thus led to larger amounts of p-ERK, a response pattern that might cause reduced IFN-γ production by B6/*lpr*-p21tg T cells. p21tg expression was also confirmed after secondary activation (0–6 h; Fig. 6G). We found no p-ERK expression differences in B6 and B6/*lpr*-p21tg T cell cultures (Fig. 7D), reinforcing our view that p21 overexpression affects *lpr* but not normal T cells.

The results indicate that, as for B6/*lpr*-p21tg T cells *in* *vivo*, p21 transgene expression regulates overexpansion, overactivation and increased IFN-γ production by B6/*lpr* T cells *in vitro*.

### Reduction of hyperproliferating effector/memory T cells ameliorates autoimmune disease manifestations in B6/*lpr* mice

**Figure 8:**
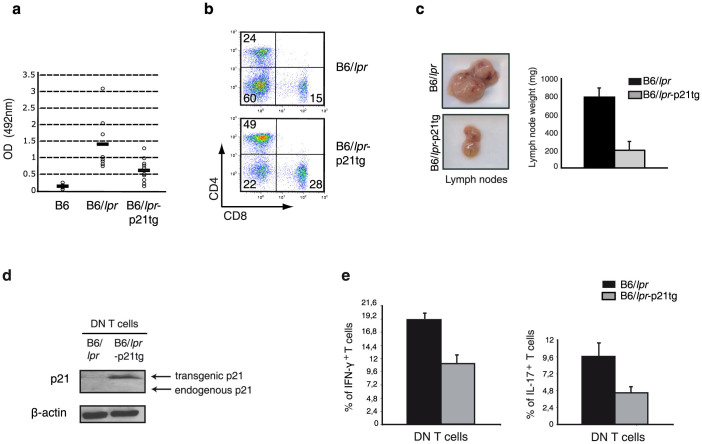
p21 overexpresson reduces autoimmune disease manifestations in B6/*lpr* mice. (a) Anti-DNA IgG levels detected by ELISA in mouse serum at 4 months of age. Anti-DNA IgG production was reduced in B6/*lpr*-p21tg compared to B6/*lpr* mice. Values show mean ± SD (*n* = 9 mice; p < 10^−4^). (b) Reduced proportions of DN T cells in B6/*lpr*-p21tg lymph nodes compared to B6/*lpr* at 5 months of age (*n* = 4 mice). (c) Reduced cervical lymph node size and weight in B6/*lpr*-p21tg females compared to B6/*lpr* at 8 months of age. Values show mean ± SD (*n* = 10 mice). (d) Western blot showed high transgenic p21 protein levels in DN T cells from 2-month-old B6/*lpr*-p21tg mice. Endogenous p21 is not expressed in B6/*lpr* or B6/*lpr*-p21tg DN T cells. β-actin was used as a loading control. (e) Intracellular staining showed reduced production of IFN-γ (left) and IL-17 (right) in B6/*lpr*-p21tg DN T cells. Values show mean ± SD (n = 4 mice; p < 10^−2^ for both cytokines).

B6/*lpr* mice show mild autoimmunity compared to the lethal lupus-like autoimmune glomerulonephritis in MRL/*lpr* mice; B6/*lpr* mice nevertheless develop a large effector/memory T cell population as well as anti-DNA autoantibodies and slight lymphadenopathy^18^. T cell-directed p21 overexpression in B6/*lpr* mice reduced not only the effector/memory T cell compartment, but also activation of IFN-γ production by these cells. As IFN-γ is indispensable for anti-DNA autoantibody production^19^, we predicted a decrease in these autoantibodies in B6/*lpr*-p21tg mice; indeed, we observed a sharp drop in anti-DNA antibodies in B6/*lpr*-p21tg mouse serum compared to B6/*lpr* controls (Fig. 8A). Although the effect of the p21 transgene leads to reduced T cell activation and associated disease manifestations, alternatively, increased p21 expression may affect disease development in a paracrine manner. As macrophage activation is critical for *lpr* disease development^42^, we examined whether these cells could be affected by the p21 transgene in such manner. Peritoneal B6/*lpr* macrophages were exposed to culture supernatants from repeatedly stimulated B6/*lpr* or B6/*lpr*-p21tg T cells. The concentration of IFN-γ in T cell culture supernatants was strongly reduced in the presence of the p21 transgene (Fig. S7). While the supernatants from B6/*lpr* T cells highly activated macrophages, as seen by high STAT1 phosphorylation and iNOS induction, equivalent supernatants from B6/*lpr*-p21tg showed lower STAT1 phosphorylation and abolished iNOS induction (Fig. S7). These data also suggest that iNOS is induced only by high IFN-γ levels, a result that was supported by exposing macrophages to different IFN-γ concentrations. Overall the results highlight the potential of p21 overexpression to control essential disease components in a paracrine fashion.

B6/*lpr* mice develop lymphadenopathy and splenomegaly, caused mainly by DN T cell accumulation. Expansion of this cell subset is evident at two months of age and increases with time. p21 overexpression led to a clear reduction in the DN T cell population in B6/*lpr*-p21tg mice (Fig. 8B). Consistent with this decrease, lymphadenopathy was notably reduced in these mice compared to B6/*lpr* controls, with greatly diminished lymph node size and weight (Fig. 8C). We detected no endogenous p21 expression in DN T cells (Fig. 8D). This lack of p21 probably explains the hyperproliferation of these cells, whereas transgenic p21 expression (Fig. 8D) reduced their hyperproliferation and hyperactivation. In agreement with the inhibition of DN cell hyperactivation, we found a profound reduction in IFN-γ- and IL-17-producing DN T cells in B6/*lpr*-p21tg mice (Fig. 8E).

As DN T cells arise from CD8^+^ T cells^43^ we examined the *in vitro* characteristics of B6/*lpr*-p21tg and B6/*lpr* CD8^+^ T cells purified from the spleens of 2-months-old mice. The data showed that after secondary stimulation B6/*lpr*-p21tg CD8^+^ T cells proliferate at much lower levels compared to B6/*lpr* CD8^+^ T cells, as tested by cell cycle and BrdU analyses (Fig. S8). In contrast to DN T cells which hyperproliferated *in vivo*, the DN cells, which were generated by CD8^+^ T cells in *in vitro* cultures^44^, accumulated as non-proliferating cells, as shown by cell cycle and BrdU analyses. The percentage of accumulated DN T cells after secondary stimulation was similar for both B6/*lpr* and B6/*lpr*-p21tg CD8^+^ T cell cultures (approximately 10%) (Fig. S8), indicating that p21 affects DN T cell expansion only *in vivo*.

These data show that T cell-directed expression of the p21 transgene had a dual effect in B6/*lpr* mice; first, it lessened autoimmune manifestations, due to the transgene effect on IFN-γ production, and second, it limited DN T cell-induced lymphadenopathy by reducing DN T cell hyperactivation and hyperproliferation.

### p21 overexpression reduces lupus-like autoimmunity and increases MRL/*lpr* mouse survival

As p21 overexpression in B6/*lpr*-p21tg T cells limited autoimmune symptoms and lymphadenopathy in B6/*lpr* mice, we tested its potential to inhibit the severe, death-inducing autoimmunity in MRL/*lpr* mice. T cell-directed p21 overexpression reduced the percentage of lymph node DN T cells as well as lymphadenopathy development (Fig. S5) in MRL/*lpr*-p21tg compared to MRL/*lpr* mice.

**Figure 9:**
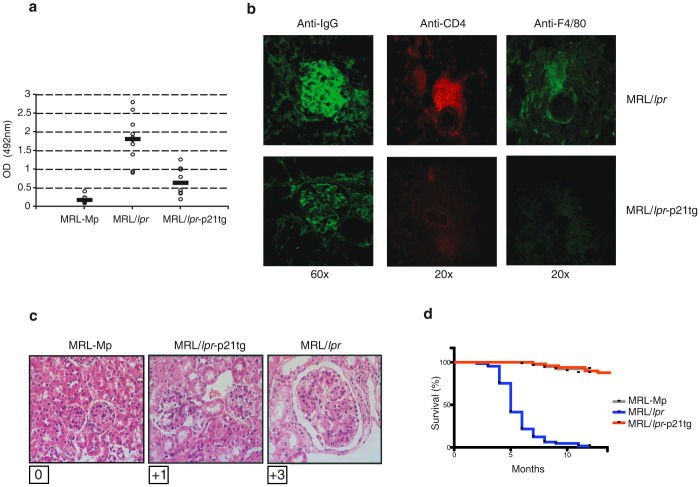
p21 overexpression reduces autoimmune disease symptoms and mortality in MRL/*lpr* mice. (a) Relative anti-DNA IgG levels detected by ELISA in serum at 4 months of age. Anti-DNA IgG production was decreased in MRL/*lpr*-p21tg compared to MRL/*lpr* mice. Values show mean ± SD (*n* = 9 mice; p < 2 × 10^−4^). (b) Immunostaining of kidney cryosections, showing reduced immune complex deposition (anti-IgG), CD4^+^ T cell (anti-CD4) and macrophage (anti-F4/80) infiltration in MRL/*lpr*-p21tg mice at 6 months of age. (c) Evaluation of glomerulonephritis grade (Berdem scale) in representative kidney sections from MRL-Mp, MRL/*lpr* and MRL/*lpr*-p21tg mice at 6 months of age showed reduced glomerulonephritis in MRL/*lpr*-p21tg than in MRL/*lpr* mice. (d) Kaplan-Meier survival curves indicate the effect of the p21 transgene on MRL/*lpr*-p21tg mouse lifespan.

MRL/*lpr*-p21tg mice showed sharply reduced (>4-fold) anti-DNA antibody levels that decreased nearly to values for MRL-Mp controls, which develop mild autoimmunity later in life (Fig. 9A). As MRL/*lpr* mice have much higher anti-DNA antibody levels than B6/*lpr* mice, p21 overexpression appeared to control the predisposition to severe autoimmunity. Compared to MRL/*lpr* mice, MRL/*lpr*-p21tg mice showed clear improvement in critical aspects of lupus-like kidney disease such as lower immune complex deposition, decreased inflammatory infiltration of CD4^+^ T cells and F4/80^+^ macrophages (Fig. 9B), and reduced glomerulonephritis (Fig. 9C). These data were corroborated by proteinuria analysis showing significantly reduced proteinuria for MRL/*lpr*-p21tg compared to MRL/*lpr* mice (1.5+ *vs* 4.0+, *n* = 10 mice per group; p < 10^−3^). This p21 transgene effect on MRL/*lpr* disease manifestations resulted in extended survival of MRL/*lpr*-p21tg mice (Fig. 9D).

These findings indicate that p21 is a potent suppressor of autoimmunity, as it can restrain autoimmune symptoms and death associated with severe lupus-like disease in the MRL/*lpr* mouse model.

## Discussion

Deficiency in p21 when combined with autoimmunity-prone backgrounds leads to severe lupus-like disease and death, suggesting that p21 moderates autoimmunity progression^5^,^6^. On the other hand, lack of p21 in normal background mice results only in mild autoreactivity^7^, questioning a possible effect of p21 in autoimmunity treatment. The aim of the present study was to explore a p21 therapeutic effect by increasing its expression levels in T cells of B6/*lpr* and MRL/*lpr* mice. We showed that enhanced p21 levels suppressed autoreactivity and DN T cell lymphadenopathy in B6/*lpr*-p21tg mice and effectively targeted severe autoimmune disease and death in MRL/*lpr*-p21tg mice and led to three principal conclusions. First, we provided direct evidence for a role of p21 in regulating autoimmunity through a p21 function on effector/memory *lpr* T cells that limits their accumulation, activity and IFN-γ production. Second, we established that preactivated but not naïve *lpr* T cells require elevated p21 expression to control activation. Finally, since normal background B6-p21tg T cells remained unaffected by increased p21, our work imparts an experimental model that specifically targets autoimmune, but not normal immune responses.

In B6/*lpr* mice, activated T cells increase with age as effector/memory or DN T cells. Fas deficiency leads to defective AICD in *in vitro* conditions but deficient T cell apoptosis has not yet been shown *in vivo* in the absence of Fas^18^. Instead, all *lpr* T cell subsets exhibit a hyperactivation state and hyperproliferate^45^,^46^,^47^, suggesting that alternative Fas functions may control *lpr* T cell proliferation^24^. T cell-specific p21 overexpression severely reduces effector/memory and *lpr* DN T cell expansion. We did not detect a role for overexpressed p21 in AICD induction, as B6/*lpr* and B6/*lpr*-p21tg T cells showed similarly defective apoptosis after secondary stimulation, and AICD was similar for B6 and B6-p21tg T cells. Therefore we ruled out the possibility that transgenic p21 reduced *lpr* DN and effector/memory T cells by promoting their apoptosis. Together with data from a previous study that showed similar apoptosis for restimulated B6 and B6 p21^−/−^ T cells^7^, these results demonstrate that p21 is not involved in typical Fas/FasL-dependent AICD events. Furthermore, our data suggest that the *in vivo* expansion of DN cells is driven by an uncontrolled cell cycle progression, which is dissociated from the absence of apoptosis.

The control of the effector/memory T cell population in B6/*lpr*-p21tg mice was the result of a reduced T cell activation accompanied by a sharp limitation of the proportion of T cells that secrete IFN-γ. These data suggest that overexpressed p21 does not act as a mere cell cycle inhibitor and may possess other properties in controlling T cell responses. Other studies have shown that p21 interferes with the NF-kB activation pathway in macrophages^11^ or controls the anergic state of chemically-induced T cell anergy^48^, independently of its cell cycle inhibitor function. Exogenous p21 did not affect the effector/memory T cell population of normal background B6-p21tg mice and did not influence their activation and IFN-γ production. These data indicate that endogenous p21 is sufficient to control normal T cells and that increased p21 levels do not influence the responses of B6-p21tg T cells. In agreement with this view, classical immunization with OVA or with vaccinia virus showed similar responses of B6 and B6-p21tg T cells. It is, therefore, here established that autoimmune *lpr* T cells require higher p21 levels in order to control their activation, as compared to normal T cells that remain unaffected by exogenous p21.

*In vitro* studies validated the above results, showing that after secondary stimulation B6/*lpr*-p21tg T cells acquired a less activated phenotype and produced less IFN-γ than those from B6/*lpr* mice. Importantly, direct induction of IFN-γ by IL-12 and IL-18, following primary stimulation and IL-2 treatment, was significantly inhibited by the p21 transgene. These findings indicate that overexpressed p21 may interfere with the IFN-γ induction pathway. The decreased proliferation of B6/*lpr*-p21tg T cells was accompanied by a reduction of CDK2 activation, suggesting that exogenous p21 may target proliferation through its effect on CDK2. However, B6-p21tg T cells also showed diminished CDK2 activity but proliferated similarly to B6 T cells. Theses results support the view that differences in CDK2 activation do not always translate into proliferation differences, as CDK2 is a dispensable target for cell cycle inhibition^49^,^50^. Therefore, in addition to CDK2 inhibition overexpressed p21 may also modulate the expansion of *lpr* T cells by altering their degree of activation. Accordingly, p21 affected ERK phosphorylation in B6/*lpr*-p21tg but not in B6-p21tg T cells. Other studies show association between p-ERK levels and IFN-γ production showing that ERK reduction leads to decreased or increased IFN-γ production by T cells^40^,^41^. We suggest that p21-dependent alteration of ERK phosphorylation may reduce IFN-γ production in restimulated B6/*lpr*-p21tg T cells, a point that will be addressed in depth in a later study.

Exogenous p21 had no effect on activation and IFN-γ production capacity of restimulated B6-p21tg T cells, corroborating the *in vivo* results. These data reaffirm the differential requirements of autoimmune and normal T cells in controlling their activation state, as p21 was similarly expressed in both cell types. Primary B6-p21tg and B6/*lpr*-p21tg T cell responses were not affected, showing that p21 overexpression is specifically targeting repeatedly stimulated *lpr* T cells. Thus, p21 overexpression does not inhibit autoimmunity through a general suppression of T cell responses, a result that is further supported by data showing that exogenous p21 does not affect T cell development in the thymus.

p21 overexpression markedly inhibited lymphadenopathy development in both B6/*lpr*-p21tg and MRL/*lpr*-p21tg mice. As DN T cell hyperactivation is directly linked to their hyperproliferation and accumulation^45^, p21 could regulate their expansion. Indeed, exogenous p21 greatly reduced IFN-γ and IL-17 overexpression by these cells, which is linked to hyperresponsiveness^51^, suggesting that p21 regulation of T cell activity could be used in therapeutic design for ALPS patients.

Overexpressed p21 compromised the capacity of effector/memory B6/*lpr*-p21tg T cells to produce IFN-γ, which is necessary for anti-DNA autoantibody and lupus-like autoimmunity development^19^,^42^. These results suggest that overexpressed p21 reduces T cell activation and IFN-γ secretion, which lead to downmodulation of anti-DNA antibody production. In addition, *in vitro* data suggest a possible paracrine effect of p21 transgene in disease development, since the differential IFN-γ secretion by B6/*lpr* and B6/*lpr*-p21tg T cells greatly affected macrophage activation and inflammatory potential. MRL/*lpr* mice develop severe autoimmune disease and suffer early mortality due to the concurrence of the *lpr* mutation and the MRL autoimmunity-promoting background. T cell-directed p21 overexpression strongly limited MRL/*lpr*-p21tg mice mortality by greatly reducing anti-DNA autoantibody production and kidney inflammatory infiltrates. Furthermore, it diminished the DN T cell numbers, as well as their potential to produce inflammatory cytokines, which is related to pathogenicity^51^. These data support a strong therapeutic role for p21 in increased autoreactive T cell responsiveness and in maintaining autoimmunity at bay.

Elevated IFN-γ levels are associated with SLE^52^,^53^, and clinical trials are currently testing the humanized anti-IFN-γ monoclonal antibody AMG 811 for lupus treatment (Amgen). In this context, our results suggesting that p21 inhibits autoimmunity by modulating IFN-γ could have therapeutic interest. General alterations in the activation machinery and reduced p21 levels in lupus patient T cells^54^,^55^ corroborate the therapeutic potential of our findings. The data also suggest that autoimmune memory T cell hyperresponsiveness can be reduced without affecting normal T cell responses, which would evidently constitute an ideal treatment for lupus and other autoimmune diseases. While therapeutic use of p21 overexpression might not appear to be an immediate option in humans, analysis of the mechanism by which high p21 levels reduce memory T cell expansion could provide a discovery platform for p21-associated factors suitable for clinical intervention. Finally, the selective effect of p21 on autoimmune but not normal T cells supports the idea of developing therapeutic approaches that do not affect normal immunity but robustly target autoimmune pathology.

## Methods

### Mice

Control C57BL/6 mice were obtained from Harlan Interfauna Ibérica; C57BL/6-*lpr* (B6.MRL-*Tnfrsf6lpr*) were from Jackson Laboratories. In C57BL/6-*lpr*-p21tg mice, generated from C57BL/6-*lpr* and C57BL/6-p21tg mice, transgene expression was restricted to T cells by the proximal Lck promoter^26^. Mice were kept in SPF conditions. All mouse experiments were performed in accordance with European Union and national regulations, and were approved by the CNB Bioethics Committee.

### Flow cytometry

Stained cells were analyzed on an LSR cytometer (Becton Dickinson). To determine memory phenotype, CD4^+^ T cells were stained with anti-CD8-PEcy7 (Biolegend), -CD4-PE, -CD44-FITC and -CD62L-APC (Beckman Coulter) antibodies. For double negative population analysis, cells were stained with anti-CD4-FITC (PharMingen), -CD8-PEcy7, -B220-APC (Beckman Coulter) and -TCR-PE (PharMingen) antibodies.

BrdU was detected with an anti-BrdU-FITC antibody (Becton Dickinson) combined with -CD4-Pecy7 (eBioscience), -CD8-SPRD (Beckman Coulter), -CD44-biot (PharMingen), Av-SPRD (Beckman Coulter), -CD62L-PE (Southern) and -TCR-PE (PharMingen) antibodies. To distinguish live from dead cells, we preincubated cells with the LIVE/DEAD stain kit (Invitrogen) according to manufacturer's instructions. Only live cells were considered for analyses.

### Cell culture

Mouse CD4^+^ spleen T cells were purified by Negative Isolation Kit (Dynal Biotech); >85% pure CD4^+^ T cells were obtained. In some experiments, naïve CD4^+^/CD44^low^/CD62L^hi^ cells were obtained by a combination of negative isolation and sorting on an Epics Altra sorter (Beckman Coulter) after staining with anti-CD44 and -CD62L antibodies. Purified naïve T cells (10^6^/ml) were stimulated *in vitro* with concanavalin A (ConA; 3 μg/ml, Sigma) in medium containing 20 ng/ml human recombinant interleukin-2 (rIL-2, PeproTech) or in medium conditioned with anti-CD28 (PharMingen; 1 μg/ml) in wells coated with anti-CD3 antibody (PharMingen; 1 μg/ml in 100 μl/well PBS). After 3 days, cells were washed and cultured in medium with 20 ng/ml IL-2 (24 h), followed by restimulation with IL-2 in combination with either ConA or anti-CD3/CD28 or IL-12 (10 ng/ml) plus IL-18 (10 ng/ml). In some cultures, the caspase (zVAD; 50 μM/ml, Bachem) was added.

Mouse CD8^+^ T cells were isolated from spleen by a negative isolation kit (Dynabeads Untouched Mouse CD8 cells, Invitrogen) and stimulated *in vitro* with ConA (3 μg/ml, Sigma) in complete RPMI, containing 20 ng/ml human recombinant interleukin-2 (rIL-2, PeproTech). After 3 days, cells were washed and cultured in medium with 20 ng/ml rIL-2 (24 h), followed by ConA restimulation for 24 h. During the last 3 hours, BrdU was added at a final concentration of 6.5 μg/ml and the cells were processed for flow cytometry and stained with the combination of anti-TCRβ, anti-CD8, anti-CD4 and anti–BrdU antibodies. Alternatively, cells were stained with anti-CD8-PE and 2 μg/ml 4′,6-diamidino-2-phenylindole (DAPI) for cell cycle analysis.

For macrophage cultures, peritonal exudate cells (PEC) were harvested from B6/*lpr* mice that were injected i.p. with 1 ml of 3% thioglycollate medium (Difco) 4 days before isolation. PEC were cultured for 2 h in RPMI 1640 without FBS and non-adherent cells were removed by washing with PBS and adherent macrophages were cultured overnight in complete RPMI 1640 with 10% FBS. Cells were incubated for 20 h in the presence of 1:2 diluted cell-free culture supernatants from B6/*lpr* or B6/*lpr*-p21tg T cells (collected 24 h after second ConA stimulation). Control cells were cultured in medium alone or stimulated for 20 h with IFN-γ (100 or 50 U/ml, Sigma).

### OVA immunization and *in vitro* stimulation

B6 and B6-p21tg mice were immunized with 100 μg OVA (Sigma-Aldrich) and CFA as previously described^7^. After 3 weeks, mice received a second OVA boost in IFA; mice were sacrificed one week later and lymph node cells were stained to quantify memory CD4^+^ T cells. In addition, CD4^+^ cells were purified from spleens of B6 and B6-p21tg mice one week after the second OVA boost using Dynabeads as above. Cells were OVA-stimulated *in vitro* (100 μg/ml; 10^5^ cells/ml; 24 h) in RPMI 1640 containing 10% FCS with irradiated splenocytes (0.5 × 10^6^/ml). Proliferation was measured using [^3^H]thymidine as above.

### Vaccinia virus infection and *in vitro* stimulation

B6 and B6-p21tg mice were immunized by i.p. injections at Day 0 and Day 8 with 1 × 10^7^ PFU of Modified Vaccinia Ankara (MVA) virus^36^. At Day 18, mice were sacrificed and erythrocyte-depleted spleen cells were resuspended in RPMI 1640 with 10% FCS and 1 μg/ml GolgiPlug (BD) and stimulated (6 h, 37°C, 5% CO_2_) with B8R_20–27_ peptide of vaccinia virus (sequence: TSYKFESV, CNB, Proteomics Facility) at a final concentration of 10 μg/ml.

### Intracellular cytokine staining

For intracellular cytokine staining, lymph node T cells or cells after primary ConA activation and IL-2 expansion were stimulated (1 h, 37°C) in medium with phorbol 12-myristate 13-acetate (PMA; 50 ng/ml) and ionomycin (2 μg/ml). Brefeldin A (GolgiPlug, 20 μl/ml; BD PharMingen) was added and cells incubated (3 h, 37°C). After surface marker staining with appropriate combinations of labeled anti-CD4, -CD8, -Thy1, -CD44, and -CD62L, cells were washed with PBS, fixed and permeabilized with Cytofix/Cytoperm (100 μl, 20 min, 4°C), washed once with Perm/Wash buffer and blocked with 150 μl Perm/Wash buffer + 1% BSA (30 min, room temperature (RT)). Anti-IL-17-PE, -IL-2-PE and -IFNγ-PE antibodies were added (all 1/100, PharMingen); permeabilized cells were incubated (20 min, 4°C) and washed twice in Perm/Wash before FACS analysis.

For the vaccinia infection experiment, splenocytes were stained for surface markers with anti-CD3-PE-CF594, -CD4-APC-Cy7, -CD8-V500 (all from BD), fixed, permeabilized (Cytofix/Cytoperm kit; BD), and stained intracellularly with anti-IFNγ-PE, and -TNFa-PE-Cy7 (from BD). Dead cells were excluded using the violet LIVE/DEAD stain kit (Invitrogen).

### *In vivo* BrdU administration

Mice were given BrdU (0.8 mg/ml; Sigma-Aldrich) in drinking water, which was prepared fresh every 2 days for a 9-day period, and BrdU incorporation was determined in lymph node T cells. Cells were stained with appropriate combinations of labeled anti-CD4, -CD8 and -CD44, -CD62L and -TCR and anti-BrdU antibody.

### DN T cell sorting

Mouse CD4^+^ T cells were isolated from spleen using the Negative Isolation Kit, and stained with anti-TCR-FITC (Pharmingen), -CD4-PE (Beckman Coulter) and -CD8-SPRD. DN T cells were separated from freshly isolated total CD4^+^ T cells on an Epics Altra sorter (Beckman Coulter).

### Proliferation assays

Proliferation was quantified by [^3^H]thymidine uptake (1 μCi/150 μl; Amersham Bioscience) in the last 16 h of culture. After cell transfer to fiber filters (Wallac) with a semi-automatic cell harvester, incorporated radioactivity was measured in a beta-plate counter (β1205 Wallac, Perkin Elmer).

To measure expression of the Ki-67 proliferation marker, cells were washed with PBS, fixed and permeabilized with Cytofix/Cytoperm (100 μl; 20 min, 4°C), washed once with Perm/Wash buffer and blocked with 150 μl Perm/Wash buffer + 1% BSA (30 min, RT). Anti-Ki-67 antibody (1/100, Abcam) was added; permeabilized cells were incubated (1 h, 4°C) and washed twice in Perm/Wash before flow cytometry analysis.

### Apoptosis assays

For cell cycle analysis, at the indicated times permeabilized cells were resuspended in 10 μg/ml propidium iodide (PI; 30 min, 37°C) and analyzed by flow cytometry. For annexin-V/PI double staining, cultured cells were stained using an anti-annexin-V antibody kit (PharMingen; 15 min, 4°C). To distinguish live from apoptotic and necrotic cells, 10 μl of 50 μg/ml PI stock solution was added before flow cytometry analysis.

### Western blot

Lysates (40 μg) of freshly isolated or cultured cells were analyzed with anti-p21 (Santa Cruz) anti-phospho ERK antibodies (Cell Signaling); anti-ERK (Cell Signaling), anti-CDK2 (Santa Cruz) and anti-β-actin (Sigma) served as loading controls. Macrophage whole cell lysates (20 μg) were probed with anti-phospho-STAT1 (Cell Signalling) and anti-iNOS (Abcam).

### Immunoprecipitation and CDK2 kinase assay

For immunoprecipitation and CDK2 kinase assay^11^, cells were lysed in buffer containing 50 mM Tris-HCl (pH 7.5), 150 mM NaCl, 0.5% NP-40, protease inhibitor cocktail and phosphatase inhibitor cocktail (Roche). Protein lysates (200 μg) were mixed with 2 μg anti-CDK2 (M2, Santa Cruz; overnight, 4°C) and incubated with pre-blocked protein G-Sepharose beads (25 μl, Invitrogen) for an additional 2 h. Anti-CDK2 immunoprecipitates were then incubated with 20 μl kinase buffer (20 mM Tris-HCl (pH 8.0), 10 mM MgCl_2_, 1 mM EGTA, 1 mM dithiothreitol, 1 mM NaF, protease inhibitor cocktail and phosphatase inhibitor cocktail), supplemented with 5 μg histone H1 (Roche), 0.5 mM ATP (Cell Signaling) and 10 μCi [γ-^32^P]ATP (3000 Ci/mmol; Perkin Elmer; 30 min, 30°C). Phosphorylated histone H1 was resolved by gel electrophoresis.

### Serological analysis

Relative levels of serum anti-DNA antibody were detected by enzyme-linked immunosorbent assay (ELISA), as previously described^42^. The 96-well plates were first coated with calf thymus DNA (2.5 μg/ml; Sigma Aldrich). IgG was detected with peroxidase-conjugated anti-mouse antibody (Jackson ImmunoResearch). Relative absorbance levels were measured at 492 nm in a Labsystems Multiskan Plus Plate Reader. Proteinuria levels were determined at 4 months of age using Albustix strips and measurements were evaluated as 1+, 2+, 3+, 4+ (Bayer, Elkhart, IN). IFN-γ levels in T cell-free supernatants were determined by FlowMetrix system (Luminex, Austin, TX)^11^.

### Kidney cryosections and immunofluorescence analysis

Kidneys were isolated, embedded in inclusion solution (Jung Tissue Freezing medium, Leica Microsystems) and frozen individually in liquid nitrogen. Cryosections (7 μm) were prepared in a cryostat (Leica CM1900), placed on Fischer slides (Fisherbrand Superfrost/Plus) and allowed to dry (2 h, RT). Sections were treated with 100% acetone (10 min, 4°C), dried (30 min, RT) and blocked with PBS + 2% BSA + 10% goat serum (45 min, RT). After washing (3 times, PBS), sections were incubated (1 h, RT) with combinations of anti-IgG-FITC, -CD4-PE (both from PharMingen), and -F4/80-FITC antibodies (Serotec). After washing (3 times, PBS) and mounting (Vectashield, Vector Laboratories), fluorescence was analyzed on a fluorescence microscope (Leica).

### Glomerulonephritis evaluation

Kidney samples were fixed overnight in 10% buffered formalin, embedded in paraffin, sectioned 4 μm thick and stained with hematoxilin- eosin (H-E). Glomerulonephritis was graded on kidney sections according to the Berden scale (62): (0) no glomerular lesions, (1) minimal thickening of the mesangium, (2) lesions with noticeable increases in mesangial and glomerular cellularity, (3) lesions characterized by the preceding conditions with superimposed inflammatory exudates and capsular adhesions, and (4) glomerular architecture was obliterated in >70% of glomeruli, and tubular cast formation was extensive.

### Survival analysis

The percentage of surviving mice over a 15-month time course was calculated by the Kaplan-Meier method using GraphPad Prism V.5.

### Statistical analysis

The Mann-Whitney U test was used for statistical analyses; *p* values < 0.05 were considered significant.

## Author Contributions

D.B. conceived the project. L.D. and C.V.M. designed and performed the experiments. R.F. and A.F. generated the C57BL/6-overexpressing p21 mice. J.M.F. analyzed and evaluated the histology data. M.E. Designed the vaccinia immunization experiment. Further contribution to the experimental work was as follows: A.B.T. to Figures 8c, 9 and S5, K.W. to Figures 2, 3, 6d and S2, G.R. to Figures 2, 3, 6f, 7c, 7g, S2, S6, S7 and S8. A.A.M. to Figures 6 and 7, M.D.P. to Figure 7g. D.B., C.M.A., L.D. and G.R. wrote the manuscript. All authors reviewed the manuscript.

## Supplementary Material

Supplementary Information Supplementary Information
