# Supplementary material for: Distinct p21 requirements for regulating normal and self-reactive T cells through IFN-γ production
Source: Sci Rep. 2015 Jan 9;5:7691. doi: 10.1038/srep07691 (PMC4287747; doi:10.1038/srep07691)
Supplement: Supplementary Information [file srep07691-s1.pdf]

Distinct p21 requirements for regulating normal and self-reactive T cells through IFN-γ production

Lidia Daszkiewicz, Cristina Vázquez-Mateo, Gorjana Rackov, André Ballesteros-Tato, Kathrin Weber, Adrián Madrigal-Avilés, Mauro Di Pilato, Arun Fotedar, Rati Fotedar, Juana M. Flores, Mariano Esteban, Carlos Martínez-A and Dimitrios Balomenos

Figure S1. Thymic T cell profile for B6, B6/*lpr*, B6-p21tg and B6/*lpr*-p21tg mice. Single-cell suspensions were prepared from 1.5-month-old B6, B6/*lpr*, B6-p21tg and B6/*lpr*-p21tg mouse thymus, and surface CD4 and CD8 expression analyzed by flow cytometry. Representative data are shown from three independent experiments.

Figure S2. p21 overexpression does not affect IL-2 expression in memory T cells. Flow cytometry analysis of intracellular IL-2 in lymph node $CD4^{+}CD44^{hi}$ T cells from 4-month-old B6/*lpr*, B6/*lpr*-p21tg, B6 and B6-p21tg mice. Data from one representative experiment of two performed, each experiment included $n = 4$ mice.

Figure S3. p21 expression in B6 and B6-p21tg T cells. Purified T cells from B6 and B6-p21tg mouse spleens were ConA-stimulated and -restimulated after an IL-2 expansion phase. Western blot analysis shows endogenous and transgenic p21 protein levels in both cell types at the end of IL-2 expansion (0 h) and at several points after ConA restimulation. β-actin was used as a loading control. The samples were derived from the same experiment and the two gels were run and processed simultaneously under the same experimental conditions.

Figure S4. Decreased *in vitro* proliferation of B6/*lpr*-p21tg T cells stimulated with anti-CD3 and -CD28 antibodies. (**A**) Purified spleen T cells from B6/*lpr* and B6/*lpr*-p21tg mice were costimulated with anti-CD3 and -CD28 antibodies (1st stimulation), IL-2-expanded, and restimulated with the same antibodies (2nd stimulation). Transgenic p21 reduced T cell responses only after secondary stimulation. Proliferation was measured by [^{3}H]thymidine incorporation after first or second challenge. Values show mean ± SD ($n = 4$). (**B**) B6/*lpr*-p21tg T cell generation was decreased compared to B6/*lpr* T cells after secondary stimulation, determined by trypan blue exclusion. Values show mean ± SD ($n = 4$). (**C**) Cell cycle analysis at 48 h after

anti-CD3/CD28 2nd stimulation showed decreased B6/*lpr*-p21tg T cell proliferation compared to B6/*lpr* T cells. Representative histograms are shown (n = 3).

Figure S5. Decreased double negative (DN) T cell accumulation and lymphadenopathy in MRL/*lpr*-p21tg mice. (**A**) Percentage of DN T cells in lymph nodes of 4- to 6-month-old MRL/*lpr* and MRL/*lpr*-p21tg mice. Values are mean ± SD (n = 10 mice; p = 4.24 x 10^{-5}). (**B**) Decreased size and weight of cervical lymph nodes in 4- to 6-month-old female MRL/*lpr*-p21tg *vs*. MRL/*lpr* and MRL-Mp mice. Values are mean ± SD (n = 10 mice; p = 1.7 x 10^{-6}).

Figure S6. Original gels for figure 7C. (**A**) Original gel for kinase assay. (**B**) Original gel for Western blot.

Figure S7. Decreased B6/*lpr* macrophage activation by B6/*lpr*-p21tg T cell culture supernatant. (**A**) B6/*lpr* peritoneal macrophages were exposed to supernatants from B6/*lpr* and B6/*lpr*-p21tg T cells that were submitted to a secondary ConA stimulation. Western blot analysis showed reduced phospho-STAT1 activation and absence of iNOS induction in macrophages stimulated by supernatants from B6/*lpr*-p21tg T cell cultures (left). Phospho-STAT1 and iNOS expression in B6/*lpr* macrophages stimulated with different IFN-γ concentrations (right). (**B**) IFN-γ concentrations in supernatants from T cell cultures after secondary stimulation were lower in B6/*lp*r-p21tg compared to B6/*lpr* supernatants. Values are obtained from T cell pools of three different mice.

Figure S8. Decreased proliferation of B6/*lpr*-p21tg $CD8^+$ T cells after secondary stimulation. (**A**) Cell cycle analysis after DAPI and CD8 staining showed reduced cell cycle progression of B6/*lpr*-p21tg $CD8^+$ T cells compared to B6/*lpr* cells, after secondary stimulation of purified $CD8^+$ T cells. DN T cells showed absence of cell cycle progression in both cases. (**B**) BrdU analysis of purified $CD8^+$ T cells 24 h after secondary stimulation. BrdU was added at

the last 3 h of stimulation. Representative plots show gated TCR^+ cells and data indicate higher BrdU incorporation in B6/*lpr* compared to B6/*lpr*-p21tg $CD8^+$ cells. DN T cells are represented as $CD8^-$ cells that do not incorporate BrdU. DN cells are generated in similar percentages after secondary stimulation of B6/*lpr* and B6/*lpr*-p21tg $CD8^+$ cells. Data are obtained from T cell pools of three different mice.

Figure S1

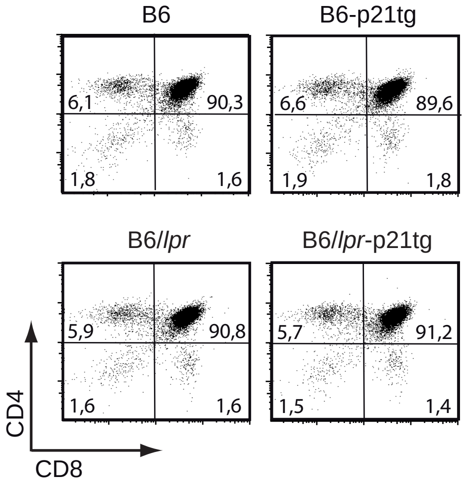
B6
6,1
90,3
1,8
1,6
B6-p21tg
6,6
89,6
1,9
1,8
B6/*lpr*
5,9
90,8
1,6
1,6
B6/*lpr*-p21tg
5,7
91,2
1,5
1,4
CD4
CD8

Figure S2

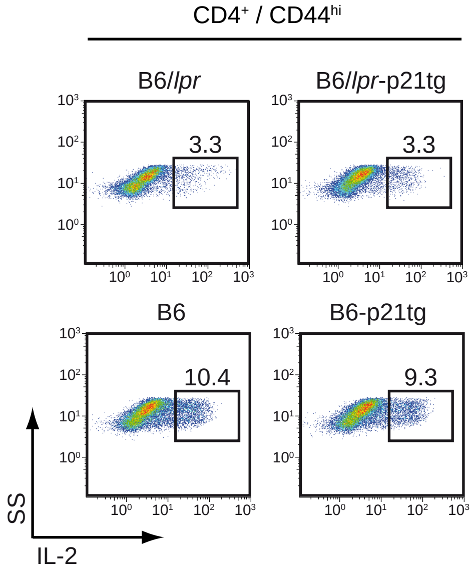

Figure S3

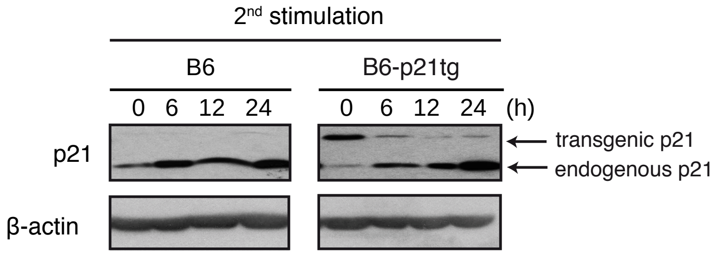
2nd stimulation
B6
B6-p21tg
0 6 12 24
0 6 12 24 (h)
p21
β-actin
transgenic p21
endogenous p21

Figure S4

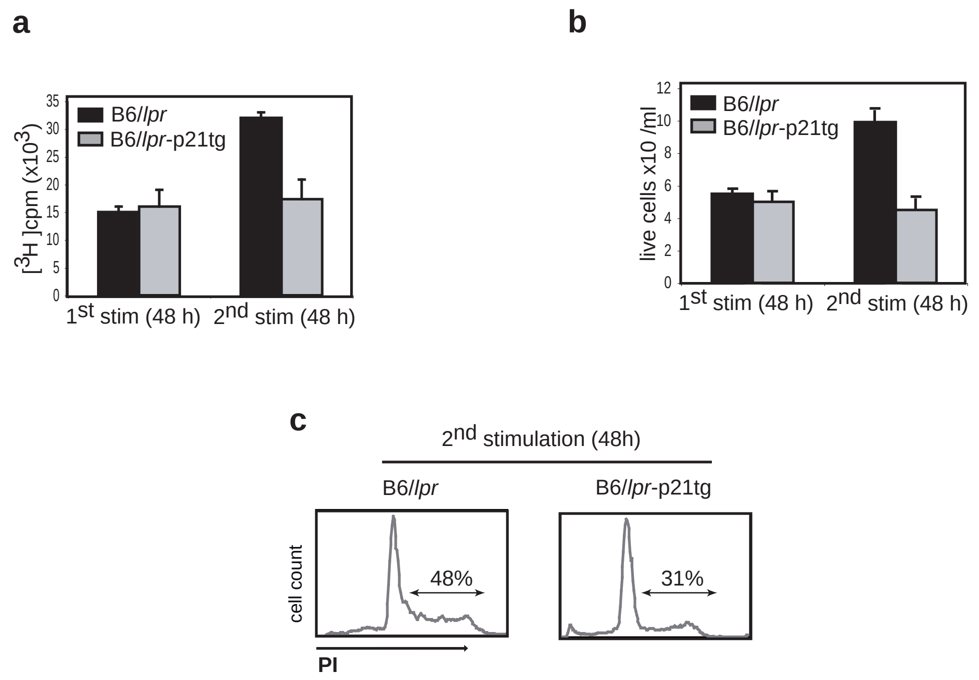
a
b
c
B6/lpr
B6/lpr-p21tg
[3H]cpm (x103)
35
30
25
20
15
10
5
0
1st stim (48 h)
2nd stim (48 h)
live cells x10 /ml
12
10
8
6
4
2
0
2nd stimulation (48h)
48%
31%
cell count
PI

Figure S5

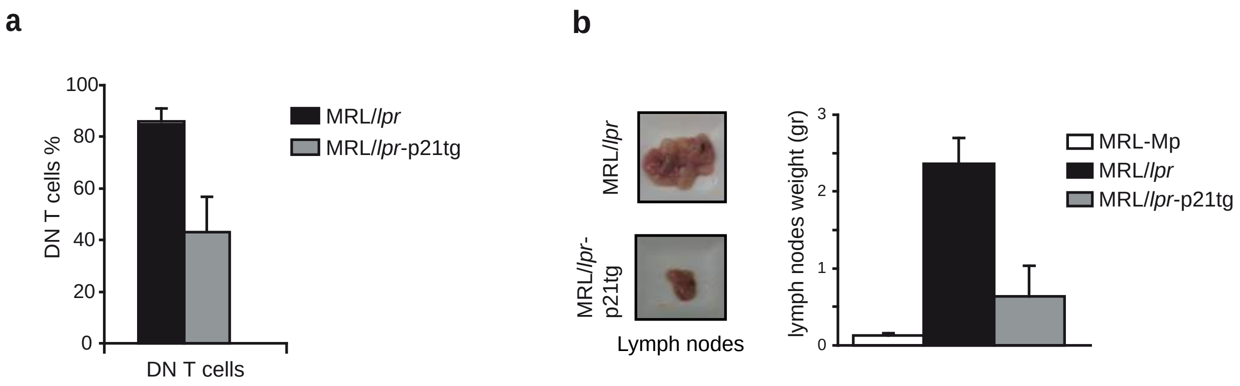
a
100
80
60
40
20
0
DN T cells %
DN T cells
MRL/*lpr*
MRL/*lpr*-p21tg
b
MRL/*lpr*
MRL/*lpr*-p21tg
Lymph nodes
3
2
1
0
lymph nodes weight (gr)
MRL-Mp
MRL/*lpr*
MRL/*lpr*-p21tg

Figure S6

a

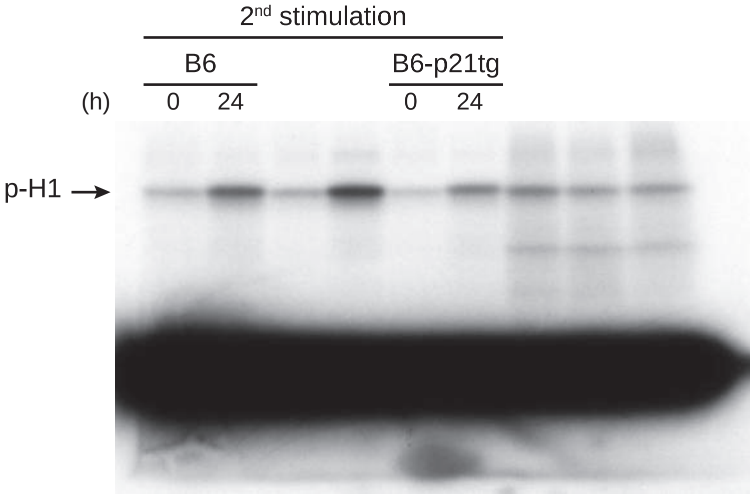
2nd stimulation
B6
B6-p21tg
(h)
0
24
0
24
p-H1

b

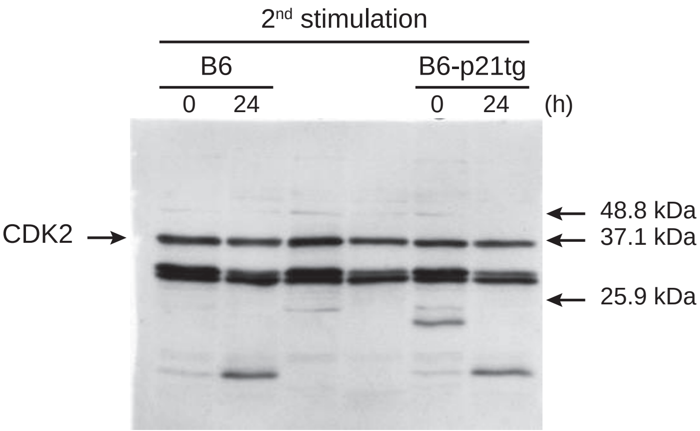
2nd stimulation
B6
B6-p21tg
0
24
0
24
(h)
CDK2
48.8 kDa
37.1 kDa
25.9 kDa

Figure S7

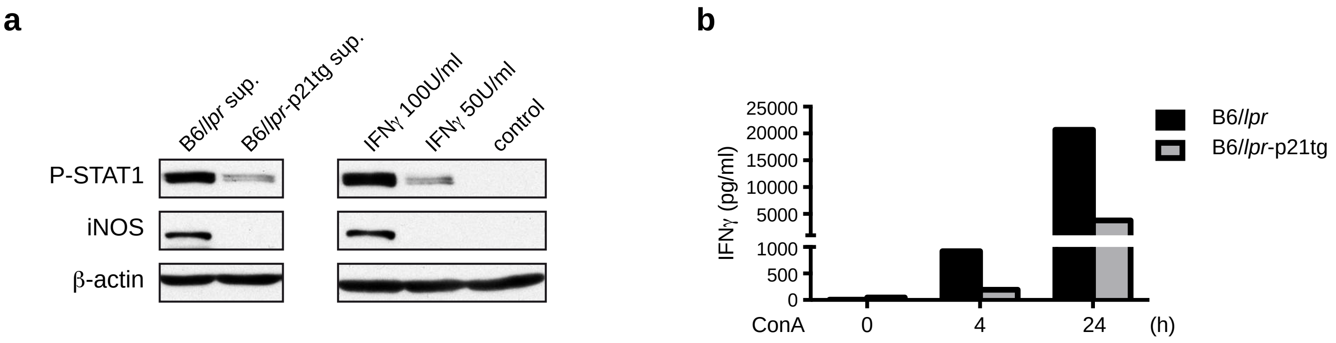
a
B6/lpr sup.
B6/lpr-p21tg sup.
IFNγ 100U/ml
IFNγ 50U/ml
control
P-STAT1
iNOS
β-actin
b
IFNγ (pg/ml)
25000
20000
15000
10000
5000
1000
500
0
ConA
0
4
24
(h)
B6/lpr
B6/lpr-p21tg

Figure S8

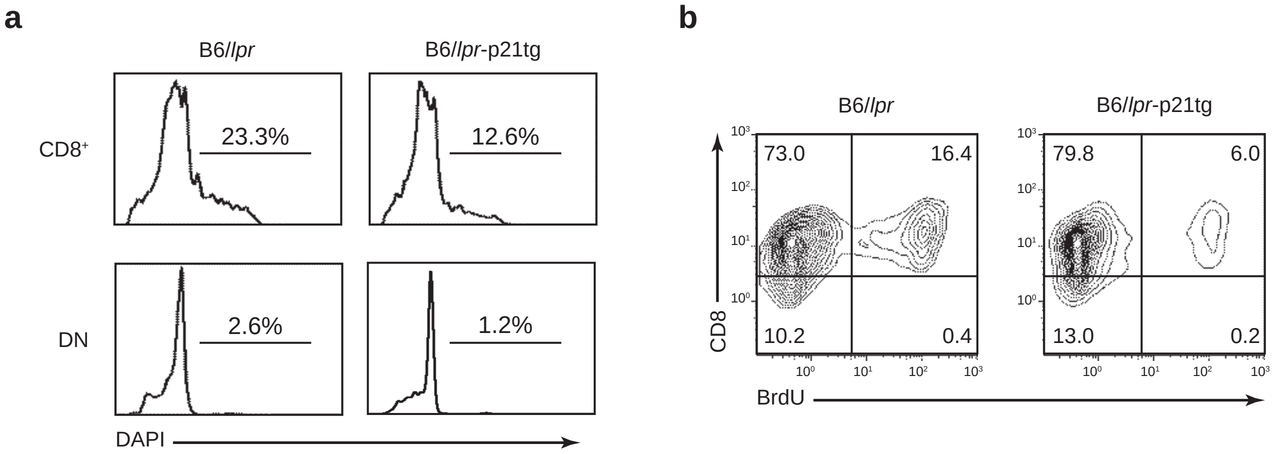
a
B6/lpr
B6/lpr-p21tg
CD8+
23.3%
12.6%
DN
2.6%
1.2%
DAPI
b
B6/lpr
B6/lpr-p21tg
73.0
16.4
10.2
0.4
79.8
6.0
13.0
0.2
CD8
BrdU
